# The Health Effects of Strongyloidiasis on Pregnant Women and Children: A Systematic Literature Review

**DOI:** 10.3390/tropicalmed3020050

**Published:** 2018-05-18

**Authors:** Matthew Paltridge, Aileen Traves

**Affiliations:** College of Medicine and Dentistry, James Cook University, Smithfield QLD 4878, Australia; Aileen.Traves@jcu.edu.au

**Keywords:** Strongyloidiasis, low birth weight, wasting

## Abstract

Strongyloidiasis is a helminth infection that remains under-researched despite its ability to cause significant illness. Women and children may be at particular risk of health consequences from this parasite. This systematic literature review aims to examine research on the long-term health effects that strongyloidiasis has in pregnant women and children. We conducted a structured search using multiple databases to collect all primary studies discussing health effects of strongyloidiasis in the aforementioned groups. The review included 20 results: 16 primary studies and four case reports. The methodological quality of studies was substandard, and there was substantial heterogeneity to the statistical analysis and outcomes assessed in the literature. Statistically significant associations were found between strongyloidiasis and low birth weight, as well as wasting. No links were found between strongyloidiasis and anaemia. Due to testing methods used in the studies, the prevalence of *Strongyloides stercoralis* in these studies was probably under-estimated. Current research is suggestive that strongyloidiasis has long-term adverse health effects on the offspring of infected mothers and in chronically-infected children. Data analysis was hindered by both methodological and statistical flaws, and as such, reliable conclusions regarding the health impacts could not be formed.

## 1. Introduction

Strongyloidiasis is a Neglected Tropical Disease (NTD) caused by the infection of a host with the soil-transmitted nematode of the *Strongyloides* genus. Humans are most commonly infected by *S. stercoralis*. However, *S. fuelleborni* has also been observed to infect humans in Papua New Guinea (PNG). *S. stercoralis* is believed to infect over 370 million people worldwide [1].

Strongyloidiasis was originally identified as a disease to look for primarily in returned travellers, refugees, or war veterans [2,3]. The life cycle of *S. stercoralis* grants it the ability to autoinfect hosts, thereby allowing the parasites to persist chronically in individuals. The parasite was believed to exist in the small intestines of hosts in a predominantly asymptomatic state, but it occasionally caused low-grade epigastric pain and diarrhoea. Studies have instead shown that intermittently symptomatic strongyloidiasis is more common than asymptomatic infection [4]. As more precise serological tests are being developed, recent data is showing that the prevalence of *Strongyloides* infections remains consistently underestimated [2,5].

There are a variety of testing methods currently utilised to detect *S. stercoralis* infection. However, they differ greatly in their accuracy [6]. The current gold standard as recognised by the Centers for Disease Control and Prevention (CDC) is seven stool samples using specialised testing techniques such as the Baermann concentration or nutrient agar plate cultures [7]. While studies typically relied on stool samples, modern serological techniques have been proven to be more accurate and practical for use in research [8].

Strongyloidiasis has also been associated with life-threatening illness. Immunosuppression allows the parasite to multiply rapidly in the small intestine and migrate through the gut to the bloodstream and other organs. This is called disseminated strongyloidiasis (DS) or hyperinfection syndrome (HS), which has a mortality rate of 85–100% from overwhelming sepsis [9]. Common conditions that cause adequate immunosuppression include HTLV-1 infection, malignancy, and exogenous corticosteroid administration [10].

While researchers have been able to shed light on the acute health effects of strongyloidiasis, the long-term consequences of this infection have not yet been fully identified. One of the main hypothesised chronic effects of strongyloidiasis is malnutrition; however, there is insufficient evidence to be certain. Milner et al. have postulated a model by which malabsorption occurs in *S. stercoralis*-infected patients, where the infection causes oedema and inflammation of the small intestine walls and prevents nutrient uptake [11]. While this is an old study that has struggled to be replicated in similar projects, a theoretical mechanism of malabsorption exists for *S. strongyloides*, suggesting that the parasite may well lead to malnutrition [12]. If this is the case, chronic strongyloidiasis has a direct clinical relevance to particular sub-groups of society that are most susceptible to harm from malnutrition, such as pregnant women, infants, and children.

Pregnant women are most likely to be affected by strongyloidiasis through two mechanisms: acute severe infection due to immunosuppression, or chronic nutritional deficiencies. Physiological changes during pregnancy cause a level of immunosuppression in the mother, placing her at an increased risk of HS or DS [13,14]. While pregnancy alone has not been observed to cause severe strongyloidiasis, corticosteroids are often administered to women when clinicians suspect a preterm delivery, and this combined effect may immunosuppress the mothers sufficiently to cause severe infection [15]. Some parasitic infections have also been known to cause anaemia during pregnancy, and theoretically *S. stercoralis* may do the same [16,17].

When looking at the risks to pregnant women, the risks to their unborn fetuses must also be considered. The offspring of infected mothers may be placed at an increased risk of harm from the effects of maternal malnutrition. Multiple studies have researched whether maternal helminth infections are a risk factor for poor pregnancy outcomes, such as intra-uterine growth restriction (IUGR) or low birth weight (LBW). While there is evidence to suggest that this may be the case, it remains unsubstantiated [18,19,20,21]. Helminth genera such as *Ascaris* or *Ancylostoma* tend to be the focus of such studies; thus there is insufficient literature to demonstrate whether strongyloides does or does not contribute to LBW as well. This is an important gap in knowledge, because if strongyloidiasis does in fact lead to LBW, this means that clinicians are not appropriately screening and treating populations in endemic areas.

Children more generally may also be affected if they become chronically infected during their childhood. Chronic diseases in childhood that produce poor growth are a particular public health concern due to the long term multifactorial consequences they have on individuals and society [22,23]. If strongyloides does cause malabsorption, this will affect the growth and development of children and leave them at an increased risk of poor health outcomes later in life. Insufficient literature is available to determine whether this is the case.

As an NTD, there is a lack of adequate research analysing the effects of strongyloides. Very few studies mention the specific consequences for pregnant women and children, and no systematic reviews have been performed studying the chronic health effects of strongyloidiasis. Those studies that do discuss chronic harm present enough theoretical and observational evidence to hypothesise that there are major health impacts on these sub-groups of the population, justifying the need for a more expansive review of the literature. This review aims to systematically summarise current evidence on the long-term effects that chronic strongyloidiasis has on pregnant women, their offspring, and infected children in general. By doing so, we hope to determine whether these sub-groups are at a potentially higher risk of harm than the rest of the population and highlight current gaps in research.

## 2. Materials and Methods

This systematic literature review was conducted and reported according to the Preferred Reporting Items for Systematic Reviews and Meta-Analyses (PRISMA) guidelines (Appendix A) [24]. The review focuses on primary studies that measured the health outcomes of strongyloides infections in pregnant women, the offspring of infected women, and children who were chronically infected during their childhood. The review has been structured according to the ‘Narrative Synthesis’ format described by Mays et al., for ease of reading and in order to easily draw conclusions between studies with different objectives and designs [25]. The review was registered with the PROSPERO International Prospective Register of Systematic Reviews (ID: CRD42017069403). Specific questions this review attempts to answer include: what are the current acute and chronic health effects that strongyloidiasis poses to these cohorts, and, what gaps remain in the literature studying chronic strongyloidiasis?

### 2.1. Search Strategy

The search strategy was based on the database sources of Medline, PubMed, CINAHL, Web of Science, Informit, The Cochrane Collaboration, Scopus, and Google Scholar. A subsequent snowball search of the reference lists of included full text articles was conducted to find further relevant sources. References were stored using EndNote X8™.

The search strategy was centred around six key concepts highlighted in Table 1: ‘strongyloidiasis’, ‘severe disease’, ‘pregnancy’, ‘infant’, ‘immune status’, and ‘eosinophilia’. ‘Severe disease’ was defined as any disease classified in the article as disseminated strongyloidiasis (DS) or hyperinfection syndrome (HS). Pregnancy included the presence of a live fetus at any week of gestation. ‘Immune status’ was used to find references to immunosuppressed populations in which strongyloidiasis may occur, such as HIV or HTLV1-infected cohorts.

Synonyms were drafted with the help of other strongyloidiasis-related literature reviews [6,26,27]. Search terms were modified to fit with the search requirements of each database used, including the use of MeSH terms for PubMed. Literature searching commenced on 26 July 2017 and was completed on 9 August 2017. The full search strategy outlining the combinations of terms used is depicted in Table 2.

### 2.2. Data Collection and Analysis

Titles and then abstracts were screened for potential inclusion. The full texts were then read to determine their eligibility according to the search criteria. If the full text could not be found, attempts were made to contact the authors or other institutions to access a full text. Articles that met the criteria were included for analysis.

A standardised spreadsheet was used to extract data from the full text articles. Data items obtained included study date, sample size, country, funding sources, ethics approval, characteristics of participants, outcomes measured, method of testing for *S. stercoralis*, statistical analysis performed, prevalence of strongyloidiasis, eosinophilia, limitations or confounding factors, and results of outcomes measured. Thematic analysis involving the simple pooling of data items was performed. Included studies and their data points were presented in both individual and aggregated tables. Due to the articles being primarily observational studies with a heterogeneous range of outcomes studied, meta-analysis was not performed.

### 2.3. Inclusion Criteria

We included all quantitative studies that tested for *Strongyloides* infections in cohorts of pregnant women, newborns, or children aged 0 to 18 years of age. If the study cohort focused on children, articles were only included if they measured the long-term effects on participants. Articles were included regardless of what outcomes they measured, such as haemoglobin (Hb) levels, neurocognitive function, or anthropometry. Types of research that were included in this review consisted of randomised control trials, observational studies, and individual case reports. Case reports were included due to the limited results that we found in our scoping searches prior to the formal literature search. By including case reports on top of the more rigorous primary literature, we can present a full landscape of current studies of strongyloidiasis in pregnant women and children.

### 2.4. Exclusion Criteria

Epidemiological studies that only commented on risk factors for infection (rather than outcomes) were excluded. Animal studies were excluded as no studies specifically looking at strongyloidiasis in pregnant animals were identified. Conference proceedings, poster presentations, and abstracts without a full text were also excluded. No language restrictions or date ranges were placed on included texts.

We hypothesised that there would be very little literature discussing srongyloidiasis in pregnant women and their children, and that many of the studies may have suboptimal methodological quality. As such, we did not exclude studies based on their methodology or statistical analysis used, as if they were found to be of generally poor quality, this would be an important limitation of current research to discuss.

### 2.5. Methodological Quality

A quality assessment of the observational studies was conducted according to a scale specifically generated for this review (Table 3). The scale is a modification of previously validated tools and used criteria from the Quality Appraisal for Cadaveric Studies (QUACS) scale first used by Smith et al., the Newcastle-Ottawa Scale (NOS), and an independent scale used in a similar systematic literature review [28,29,30]. QUACS and NOS have been validated as accurate tools to use in observational studies; however, specific items were added to the scale to make it more applicable for assessing the studies included in this review [31]. Due to the heterogeneous nature of methodologies and results used, we refrained from providing a score to assess and compare quality.

Case series were assessed for quality according to the Joanna Briggs Institute Critical Appraisal Tool: Checklist for Case Reports [32]. The risk of bias evaluations was not used to exclude studies from data synthesis but rather would be utilised to comment on the current gaps in literature and accuracy of results.

## 3. Results

Database and reference list searching returned 1666 unique results, of which 94 were considered eligible for full-text review. The full texts of 29 sources could not be found, even after attempts were made to contact authors, libraries, and institutions to access a copy. These studies were decades old, focused on other parasites, and from the citation information available did not indicate that they would add further use to this review or significantly change the conclusions. This left 65 full-text articles that were read in full. Based on our inclusion and exclusion criteria, 45 articles were then excluded. Thirty-nine full texts were excluded as they were either found to be irrelevant or were solely epidemiological studies and did not measure health outcomes of their participants. Six studies were found to be review articles and were subsequently excluded. This left a total of 20 studies for inclusion.

Sixteen articles were sourced from electronic library databases, while the remaining four studies were individually added from the Google Scholar search; no further articles were added after reviewing the reference lists of included full-texts. Sixteen studies were original research papers while four articles were individual case reports that discussed *Strongyloides* in pregnant women. Countries in which studies were performed included Australia (1), France (1), Ghana (2), Guatemala (1), India (1), Kenya (1), Nigeria (1), Papua New Guinea (3), Peru (2), Tanzania (1), Thailand (2), Uganda (2), Unites States of America (USA, 2) and Venezuela (1). Publication dates of the articles ranged from 1989 until 2015. The outcomes of the search strategy are summarised in Appendix B. For the purpose of clarity, the research papers will be discussed separately to the case studies.

### 3.1. Study Characteristics

Participant demographics included pregnant women (3), pregnant women and their newborn children (5), or only infected children (8). Participants were mostly from low socio-economic environments, and hygiene was typically stated as poor in the studies. Only one study focused specifically on *S. stercoralis* infections, and two studies focused on *S. fuelleborni*. The majority of studies either surveyed all helminth infections in their cohorts, or their main focus was on other helminths, malaria, or HIV, and happened to include data on strongyloidiasis. The study settings included in-hospital environments (3), community settings (10), or antenatal and postnatal clinics (3) (Table 4).

### 3.2. Quality Assessment

**Figure A3:**
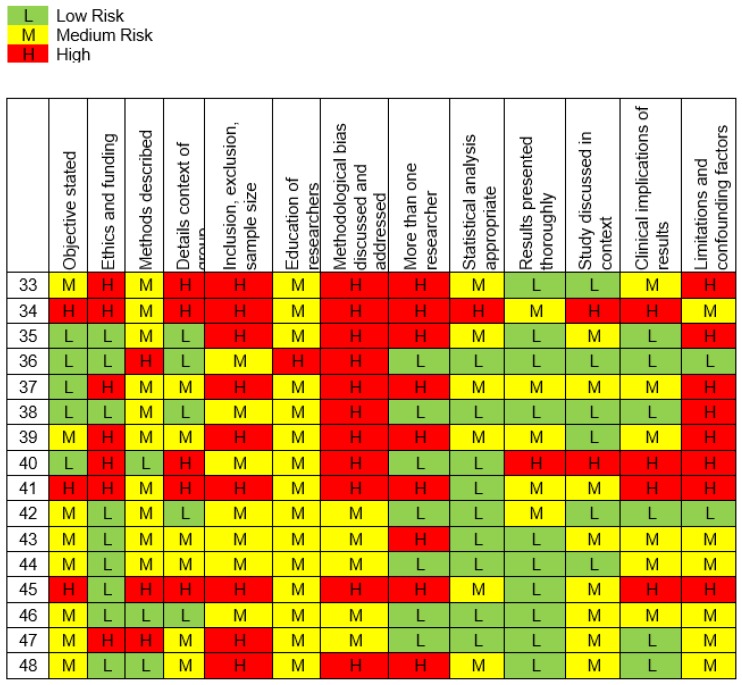
Quality assessment scale.

Overall, the methodological rigour of the literature was of a low quality. With regard to the prospective studies, 16 were cohort studies while only one was a case-control study; there were no randomised controlled trials included. The studies were found to vary widely in their study designs, attempts to control bias, and outcomes measured (Appendix C, Figure A3).

Nine studies used logistic regression, while five studies use chi-square analysis. The majority of studies did not consider or report potential bias being present in their studies and took limited steps to prevent this from affecting their results. Many studies did not adequately discuss their inclusion and exclusion criteria, sample sizes, or the involvement of researchers in data collection. The cohorts were often arbitrarily chosen and not adequately matched. Many of the studies were quoted to be using data from other larger trials without discussing any potential bias in these results. The majority of studies only reported data selectively and did not specify the non-significant results.

**Figure A4:**
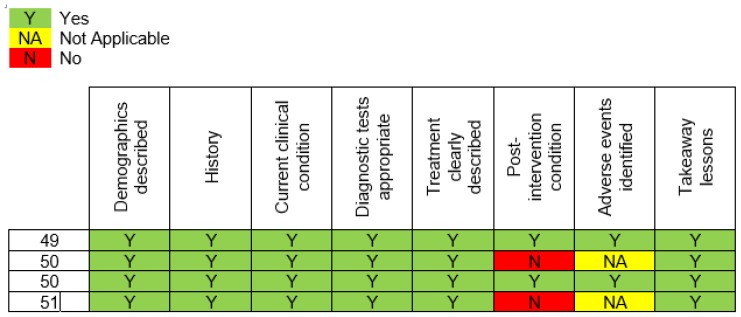
Quality assessment according to JBI Critical Appraisal Tool: Checklist for Case Reports.

Case reports were overall of a high quality and comprehensively discussed the clinical relevance of their patients (Appendix C, Figure A4).

### 3.3. Risk of Bias of Included Studies

Generally, the literature did not appropriately comment on potential limitations or confounding factors to their research. Some commonly mentioned limitations amongst studies included small sample sizes, small prevalence data of *S. stercoralis*, inappropriate testing methods, and inability to follow-up with included participants.

### 3.4. Prevalence

The prevalence of *S. stercoralis* in the studies was typically low, with a mean prevalence of 12.3% and the median prevalence being 6%. Studies with mixed urban and rural cohorts found that the prevalence of strongyloidiasis was higher in participants from rural areas. A few studies noted that their prevalence data was unreliable due to using suboptimal testing methods that have been proven to be inaccurate for detecting *S. stercoralis* [6].

### 3.5. Method of Testing

All the studies included in this review used various methods of stool-sample analysis to determine the rates of *S. stercoralis* infections in their cohorts. Methods included the Baermann (3 studies), Kato-Katz (4), Ritchie (1), formol-ether concentration (2), simple smear technique (1), volume dilution method (1), or were unspecified (4). No studies utilised serological testing. Single samples with limited follow-up were used, rather than the serial testing of multiple specimens.

### 3.6. Effects on LBW

Four studies measured the effect that *S. stercoralis* infection in pregnant women had on the birth weight of their offspring [36,38,47,48]. Two studies, one in Thailand and one in Tanzanian HIV-infected mothers, found odds ratios of 4.93 (95% CI 1.47, 16.50) and 4.23 (95% CI 1.24, 14.41), respectively, that LBW was caused by strongyloidiasis (Table 5) [36,38]. One study in Ghana found an odds ratio of 2.1 (95% CI 0.97, 4.49) for LBW, small for gestational age (SGA), or preterm delivery [48]. The fourth study found a non-significant increased risk of IUGR; the study attributed a low prevalence of strongyloidiasis to the failure to achieve a statistically significant result [47]. IUGR was predominantly seen in malnourished women who had strongyloidiasis, and the study authors hypothesised this as a possible cause rather than the helminth itself. None of the studies measured the length of duration of *S. stercoralis* infection in the pregnant mother, or the intensity of larval output as an indicator of the severity of the infection.

### 3.7. Anthropometry

Five studies analysed whether there were long term effects of *Strongyloides* infections on the nutrition and growth of infected children (Table 6) [39,40,41,42,46]. Three studies found that strongyloidiasis was associated with decreased weight-for-height or weight-for-age z-scores. However, not all of these were statistically significant [39,40,46]. These measurements are more typically used to determine wasting, which is an acute indicator of malnutrition, rather than the more chronic indicator of stunting. One study found a statistically significant relationship between strongyloidiasis and decreased height-for-age z-score (*p* < 0.01), which is used to measure stunting [39]. One study found that at 30 months of age, children with strongyloidiasis had a decreased head circumference (*p* = 0.002); this same study did not find any significant link to other anthropometric measures. One study found no statistically significant relationships between stunting, wasting, and *S. stercoralis* infections, but did note that the intensity of infection was associated with decreased weight-for-age and weight-for-height z-scores, within the *Strongyloides*-infected population (*p* = 0.02, 0.016 respectively) [41]. Another article found that children infected with strongyloidiasis were substantially more likely to suffer from marasmus or kwashiorkor when compared to non-infected children (*p* = 0.001) [40]. The majority of studies also found that polyparasitism was more strongly associated with lower z-scores of all the anthropometric measures when compared to solely *S. stercoralis* infection.

Generally, consensus from the studies was that malnutrition either predisposes participants to *S. stercoralis* infections or chronic strongyloidiasis may cause malnutrition. Due to their study designs, firm conclusions could not be made to determine whether strongyloidiasis is a risk factor or consequence of malnutrition. This issue becomes further complicated when considering that strongyloidiasis was seen more commonly in populations of lower socio-economic status, as malnutrition is also more commonly observed in these groups.

### 3.8. Strongyloidiasis and Anaemia

Five studies measured whether *S. stercoralis* contributed to maternal anaemia [33,35,43,45,46]. One study found that helminth infections were a predictor of iron-deficiency anaemia.However, the helminths were not differentiated in the results so conclusions cannot be made about the effects of *S. stercoralis* [33]. The other four studies found no relationship between strongyloidiasis and anaemia.

### 3.9. Case Reports

Four case reports were found as part of the literature search that fulfilled our inclusion criteria (Table 7) [49,50,51,52]. All four cases describe women who presented at varying stages of gestation with predominantly gastrointestinal or respiratory symptoms. Two cases were classified as HS, one as DS, and one was symptomatic but non-disseminated. Corticosteroids preceded two of the cases, and in both of these cases the patients were found to have either HS or DS. Two women had HTLV-1 co-infections, which has been previously noted as a common occurrence. Two women required ICU admissions, and one patient died from cardiorespiratory arrest secondary to septic shock. Fetal demise also occurred in the patient who passed away [48].

All patients were treated with ivermectin. Of the three women who survived, all underwent spontaneous vaginal births to healthy babies, with no complications. They made a full recovery from the infection, although one mother re-presented a year later, again pregnant and suffering from gastrointestinal symptoms [50]. She was found to have been re-infected with *S. stercoralis*.

## 4. Discussion

This review aimed to summarise current literature that analysed the long-term health outcomes of strongyloidiasis on pregnant women, their offspring, and children. Our findings suggest that there are enduring consequences for children that are either born to infected mothers, or who are chronically infected early in their development. To our knowledge, this is the first systematic literature review that attempts to determine the possibility of chronic health effects caused by strongyloidiasis in these subgroups.

The small number of studies that investigated the birth outcomes of newborns with infected mothers found that there is an association between strongyloidiasis and LBW. The 95% confidence intervals of these studies were large, despite always showing positive associations with LBW. Research has consistently recognised LBW to have lasting impacts on the morbidity and mortality of these newborns, as per the Barker and Brenner hypotheses [53]. The reliability of strongyloidiasis causing LBW is still questionable, as studies were conducted in developing countries with participants that had a range of other comorbidities (such as HIV). Therefore, further research and analysis of this potential risk factor is required. If strongyloidiasis is confirmed to cause LBW, the infection should be treated like other known risk factors for LBW, and women should receive appropriate prenatal screening and treatment [54].

Studies that measured the anthropometry of infected infants and children generally agreed that strongyloidiasis did result in a negative impact on their growth. Wasting and stunting are long-term detriments to the wellbeing of children, and research has established that they lead to increased medical comorbidities, reduced schooling, and reduced economic productivity [55,56]. The main effects of strongyloidiasis were only seen in the more acute measurement of wasting, rather than the more chronic indicator of stunting, thus the clinical implications of this finding are less certain. However, a study conducted by Richard et al. found that wasting was associated with stunting and the long-term effects that go with this [57]. Therefore, strongyloidiasis is likely to have clinically significant impacts on the health of people infected during childhood. Ivermectin should be used as part of existing community and school-based deworming initiatives in endemic areas, to prevent the enduring consequences of wasting.

The assessment of strongyloidiasis affecting the growth of children is affected by the small number of included articles that commented on these measurements. Studies also did not publish their full results lists, and the differences in cohort characteristics were large. Epidemiologically, the studies were unable to determine if strongyloidiasis was more common in malnourished children or if the disease process itself caused malnutrition. As the studies were cross-sectional, no studies looked at whether children had been chronically infected with strongyloidiasis. In order to confidently conclude that *S. stercoralis* does in fact cause deficiencies in growth and therefore have direct clinical consequences, longitudinal studies of affected participants with larger sample sizes need to be performed.

Several studies noted a range of common epidemiological risk factors which may lead to a greater risk of infants and pregnant women contracting this infection. Absence of footwear, other household members already being infected, and poor sanitation facilities, were all emphasised in the studies as potential risk factors. This poses another challenge to public health strategies, as newborns can quickly become chronically infested from their mothers or family members. Even if strongyloidiasis does not cause LBW, maternal infection can still cause chronic health effects due to the high likelihood of passing on their infection early in childhood and causing malnutrition, wasting or stunting. Interventions targeting water, sanitation, and hygiene (WASH) may provide a solution to reducing these epidemiological risk factors and therefore the long-term consequences of strongyloidiasis [58].

Based on the review findings, anaemia should not be considered a potential complication of *S. stercoralis* infections. This is in contrast to other helminths such as hookworm, which have been more strongly linked with anaemia [59]. Studies that have observed the clinical manifestations of strongyloidiasis in different cohorts have produced conflicting results regarding anaemia [60,61]. The primary reason why we are still not sure whether *S. stercoralis* causes anaemia is because both are typically common in under-nourished, socio-economically poorer populations with increased health comorbidities. This makes the task of attributing anaemia to the helminth difficult.

There is a paucity in literature looking at the effects of *S. stercoralis* infection on pregnant women, their offspring, and infected children. The only literature that could be found that mentions pregnant women with severe infection were case studies; no prospective studies could be found in which pregnancy was researched as a potential risk factor of HS or DS. Likewise, very little information currently exists to determine whether *S. stercoralis* infection in the mother is an independent risk factor for LBW, SGA, IUGR, or preterm delivery. As there were so few studies found analysing these variables, this may suggest a publication bias is present which has inflated the health effects strongyloidiasis has on these populations.

Although only a few case reports exist that discuss strongyloidiasis in pregnancy, the ones found in this review showed that this infection can cause severe or ultimately fatal complications. Pregnant women are already immunosuppressed and thus may be at a higher risk of hyperinfection syndrome. Clinicians must currently rely on individual cases for information on the possible disease course of strongyloidiasis or look elsewhere to different population groups. Corticosteroids preceded 50% of the onset of HS, although the sample was small. In areas endemic to *S. stercoralis*, women giving preterm birth or who are immunosuppressed are at risk of these severe complications and should be screened accordingly.

There is reason to believe that the prevalence and therefore the health effects of strongyloidiasis is underestimated in the current literature. The majority of studies only included *S. stercoralis* as one of many parasites tested. As the researchers were not directly focusing on strongyloidiasis they accordingly did not use accurate diagnostic tests and therefore are likely to have missed a significant amount of *S. stercoralis* infections in their participants. Even amongst stool samples, the gold standard of seven serial samples was not performed [1]. This may have had the potential to understate the longitudinal consequences strongyloidiasis had on children and thereby prevented studies from achieving statistical significance. This failure to achieve significance and the substantially low prevalence data compared to other helminths may have resulted in unpublished data, creating a relative publication bias in the literature reviewed. Healthcare providers would benefit from more accurate prevalence data in order to appropriately manage these subgroups. Therefore, the convenient and reliable serological tests should be used in further studies on strongyloidiasis.

If strongyloidiasis does indeed lead to adverse health effects in infants, this has direct clinical implications for endemic areas. This review strengthens arguments for increased screening and treatment of pregnant women to confer the best possible outcome for their children. Ivermectin has been proven to be safe in pregnancy, despite not currently being used due to its assigned pregnancy category of B3 [62]. The clinicians in all four case reports used ivermectin and no complications were observed. It is the opinion of the authors that there is enough favourable evidence to support the use of ivermectin in pregnancy, particularly given the significance of the consequences to mothers and children. Health practitioners in endemic areas would benefit from further clarification of whether this drug is appropriate for use in pregnant women.

The findings outlined in this review need to be considered with caution, as only a small number of studies have currently looked at these effects and their methodological quality may be considered suboptimal. While some studies did achieve statistical significance in their findings, they often occurred in small samples, in participants with comorbidities such as HIV or malnutrition, and in countries in developing nations; thus, their findings cannot be generalised.

### Limitations

A number of limitations to this review have been identified and must be taken into consideration when analysing the findings. Studies reviewed used varying methods of statistical analysis, which were presented and compared despite some results not being statistically significant or insufficiently powered. Many studies were conducted decades ago in vastly different contexts, using less accurate testing methods. It was unfortunate that some studies aggregated the results of *S. stercoralis* with other helminths such as hookworm; these were still included in the review. A significant proportion of our included full-texts also could not be found. Meta-analysis could not be performed and thus only simple pooling of results was possible. Due to the marked variation in the outcomes assessed by different studies, the findings were compared between cohorts that varied dramatically in their characteristics. Because of these limitations, a level of bias in the findings was unavoidable, despite taking steps to ensure transparency and academic rigour.

## 5. Conclusions

In conclusion, current research is suggestive that maternal strongyloidiasis is a risk factor for LBW. However, a lack of literature and sub-optimal study designs prevents this from being a certainty. Chronic infection in childhood is most strongly associated with wasting and may potentially lead to stunting. Due to similar issues, current research is unable to ascertain whether strongyloidiasis leads to malnutrition or is just more commonly found in the malnourished. The strongest conclusion gleaned from this literature review was that the prevalence of strongyloidiasis was very likely underestimated, due to the methods of testing and lack of focus on this specific helminth in the study designs. In order to truly determine whether pregnant women, their offspring, and infected children are in fact susceptible sub-groups of the population, further longitudinal research utilising modern serological techniques and control groups is required.

## Figures and Tables

**Table 1:** Concepts and synonyms used in search strategy.

#	Concept	Key Words
1	Strongyloidiasis	Strongyl * OR Anguillulose
2	Severity of disease	Disseminat * OR Hyperinfect * OR Severe OR Fatal OR Mortality OR Morbidity OR Death *
3	Pregnancy	Pregnan * OR Mother * OR Matern * OR Antenat * OR Natal OR Perinat *
4	Infant	Neonat * OR Newborn OR Infant * OR Baby * OR Fetus * OR Foetus * OR Fetal OR Preterm OR Child OR Prematur * OR Low Birth Weight OR LBW OR Birth Weight OR Intrauterine Growth Restriction OR IUGR OR FGR OR SGA
5	Immune status	Immunocompromised OR Tumour OR Cancer OR Haematolog * OR Lymphom * OR Leukaem * OR Neoplas * OR Malignan * OR HIV OR HTLV1 OR Rheumat * OR Diabet * OR Transplant * OR Steroid * OR Corticosteroid * OR Immunosuppress * OR Glucocorticoid * OR Sepsis
6	Eosinophilia	Eosin *

* keywords were truncated with asterisks added, to locate all forms of the word during the literature search.

**Table 2:** Combinations of key words used in search strategy.

Number Search	Combination
1.	1 + 3
2.	1 + 6
3.	1 + 2 + 3
4.	1 + 2 + 6
5.	1 + 3 + 4
6.	1 + 3 + 5
7.	1 + 3 + 6
8.	1 + 4 + 6
9.	1 + 5 + 6

**Table 3:** Quality assessment tool used for observational studies.

	Low Risk	Medium Risk	High Risk
Objective stated	Aims and objectives fully described with reasons for why they are important	Aims and objectives described, no reasons given for having these aims	Aims and objectives not fully described
Ethics and funding	Mentioned, no conflicts	Mentioned, potential conflicts of interest	Not mentioned or conflicts of interest
Methods described	Methods discussed and are reliable	Methods discussed, but may not be reliable	Methods not fully discussed
Details context of group	Participant characteristics outlined with discussion of how an accurate sample was ensured	Participant characteristics outlined	Participant characteristics not fully outlined
Inclusion criteria, exclusion criteria, sample size	Fully described with reasons given	Fully described	Not adequately described
Education of researchers	Education given, researchers have appropriate experience or qualifications	Education given, experience or qualifications not mentioned	Education and experience is not discussed
Methodological bias discussed and addressed	Efforts made to identify and solve potential bias	Mention of potential bias in methodology	No mention of bias in methodology
More than one researcher	More than one researcher	N/A	Only one researcher
Statistical analysis appropriate	Multivariate logistic regression is used	Chi square analysis is used	Any other form of analysis is used
Results presented thoroughly	Results fully and accurately described	Only partial results given	Important results omitted or not thoroughly described
Study discussed in context	Results analysed according to other studies	Results are analysed, some mention of current context	Results analysed with no mention to other research
Clinical implications of results	Direct clinical application of results is discussed	Mention of clinical relevance is made	No mention of clinical implications of results
Limitations and confounding factors	Study discussed limitations and confounding factors comprehensively	Some discussion of limitations and confounding factors	No discussion of limitations or confounding factors

N/A: not applicable.

**Table 4:** Study Characteristics.

Author, Year, Country	Study Design	Participant Characteristics	Sample Size	Length of Review	Setting	Prevalence
Baidoo et al., (2010), Ghana [33]	Prospective observational cohort study	Pregnant women	108	12 months	Community	2%
Barnish et al., (1989), Papua New Guinea [34]	Prospective observational cohort study	Children <5 years	12	NR	Community	63%
Cabada et al., (2014), Peru [35]	Prospective observational cohort study	Amazonian clan members, all ages	215	NR	Community	6%
Mangklabruks et al., (2012), Thailand [36]	Prospective observational cohort study	Newborns followed from antenatal clinic visits	2184	1 year 9 months	Antenatal and postnatal clinics	0.8%
Dada-Adegbola et al., (2004), Nigeria [37]	Prospective observational cohort study	Children <5 years with diarrhoea	227	NR	Hospital	5.3%
Dreyfuss et al., (2001), Tanzania [38]	Prospective observational cohort study	HIV-infected pregnant women and their newborns	822	NR	Antenatal and postnatal clinics	1.78%
Egger et al., (1990), Thailand [39]	Prospective observational cohort study	Children 3–8 years	343	NR	Community	25.4%
Herrera et al., (2006), Peru [40]	Prospective observational case-control study	Community members <20 years	100	1 month	Community	NR
King et al., (2004), Papua New Guinea [41]	Prospective observational cohort study	Children <5 years	179	4 months	Community	27%
LaBeaud et al., (2015), Kenya [42]	Prospective observational cohort study	Mothers and their infants <3 years	545	3 years	Community	NR
Muhangi et al., (2007), Uganda [43]	Prospective observational cohort study	Pregnant women	2507	1 year 7 months	Hospital	12.3%
Nampijja et al., (2012), Uganda [44]	Prospective observational cohort study	Mothers and their infants <15 months	983	2 years	Antenatal and postnatal clinics	13%
Phuanukoonnon et al., (2013), Papua New Guinea [45]	Prospective observational cohort study	Pregnant women	201	1 year 5 months	Community	3%
Verhagen et al., (2013), Venezuela [46]	Prospective observational cohort study	Children 4–17 years	390	1 year 6 months	Community	7.9%
Villar et al., (1989), Guatemala [47]	Prospective observational cohort study	Mothers and their newborns	14,914	1 year 9 months	Community	0.4%
Yatich et al., (2010), Ghana [48]	Prospective observational cohort study	Mothers and their newborns	746	2 months	Hospital	3.9%

NR: not reported.

**Table 5:** Methodology and outcomes of *Strongyloides* infections.

Study	Only *S. stercoralis* Is Assessed	Results Are Aggregated	Testing Method for *S. stercoralis*	Statistical Analysis	Results
33	No	Yes	Stool; formol-ether concentration method	Chi-square test	Helminth infections are a predictor of iron-deficiency anaemia in pregnant women
34	No	No	Stool; not specified	Correlation coefficient	Heavy infection predisposes to poor growth
35	No	No	Stool; Kato-Katz method	Chi-square test	High rates of anaemia and malnutrition in children Helminth infections not associated with these outcomes *Strongyloides* was not managed by treatment
36	No	Yes	Not specified	Multivariate logistic regression	Odds ratio of 4.93 of *Strongyloides*/hookworm infection in pregnancy causing LBW (95% CI 1.47, 16.50)
37	Yes	N/A	Stool; formol-ether concentration methods	Logistic regression	Higher rates of malnourished in *Strongyloides*-infected children Malnutrition may increase the risk of contracting *Strongyloides*
38	No	No	Stool; Kato-Katz method	Multivariate logistic regression	Odds ratio of 4.23 for *Strongyloides* causing LBW (95% CI 1.24, 14.41)
39	No	No	Stool; simple smear technique	Chi-square test	Lower mean height-for-age z-score (*p* < 0.01)
40	No	No	Stool; Baermann method	Multivariate logistic regression	Malnutrition more common in *Strongyloides* infections No relationship between *Strongyloides* and anthropometry
41	No	No	Stool; volume dilution method	Logistic regression	*Strongyloides* associated with decreased weight-for-age z-score (*p* < 0.05) Not associated with weight-for-height z-score (*p* < 0.05)
42	No	No	Stool; Ritchie method	Logistic regression	*Strongyloides* at 30 months is associated with decreased head circumference (*p* = 0.002)
43	No	No	Stool; Kato-Katz method	Logistic regression	No relationship between *Strongyloides* and anaemia
44	No	No	Stool; Kato-Katz method	Logistic regression	Negative impact on language function of infants (*p* < 0.05) Non-significant impact on gross motor, sociocognition, and self-care
45	No	Yes	Stool; not specified	Chi-square test	No relationship to anaemia
46	No	No	Stool; Baermann and Kato-Katz methods	Multivariate logistic regression	No relationship to anaemia Non-significant relationship between weight-for-age and BMI-for-age
47	No	No	Not specified	Multivariate logistic regression	Increased risk of IUGR Malnourished women with *Strongyloides* most at risk
48	No	No	Stool; Baermann method	Chi-square and *t*-test	Malaria co-infection had higher rates of pre-term delivery, small-for-gestational-age, and LBW (*p* < 0.05)

N/A: not applicable.

**Table 6:** Anthropometric changes associated with *S. stercoralis* infections.

Study	Weight-for-Age z-Score	Weight-for-Height z-Score	Height-for-Age z-Score	Head Circumference z-Score
39	NR	−1.01 (*p* = NS)	−2.03 (*p* < 0.01)	NR
40	Positive association (*p* = 0.045)	NR	No association (*p* = 0.24)	NR
41	No association	No association	No association	NR
42	No association	No association	No association	−1.69 (*p* = 0.002) at 30 months
43	NR	−0.24 (*p* = NS)	NR	NR

NR: not reported.

**Table 7:** Summary of case reports.

Author, Year, Country	Country of Origin, Gestation	Presenting Complaint	HS or DS?	Corticosteroids Administered	Treatment	Outcome
Buresch et al., 2015, USA [49]	Haiti, 25 weeks	Chest pain, dyspnoea, copious bilious vomiting	HS	Betamethasone 12 mg, 2 doses 24 h apart	Ivermectin	Septic shock, SIRS, cardiopulmonary arrest, fetal demise
Heaton et al., 2002, USA [52]	Ethiopia, 9 weeks	Diarrhoea, epigastric pain, vomiting	None	None	Ivermectin 200 µg/kg	SVB at term, cleared of infection
Malézieux-Picard et al., 2016, France [50]	Burkina Faso, 32 weeks	Abdominal pain, anorexia, constipation, weight loss	HS	Betamethasone 12mg stat	Ivermectin 200 µg/kg/day for 3 days	SVB, recovered from infection
Prasad et al., 2016, India [51]	India, 39 weeks	Cough, watery diarrhoea	DS	None	Ivermectin 12 mg	SVB, cleared of infection

SVB: spontaneous vaginal birth.

**Figure A1:**
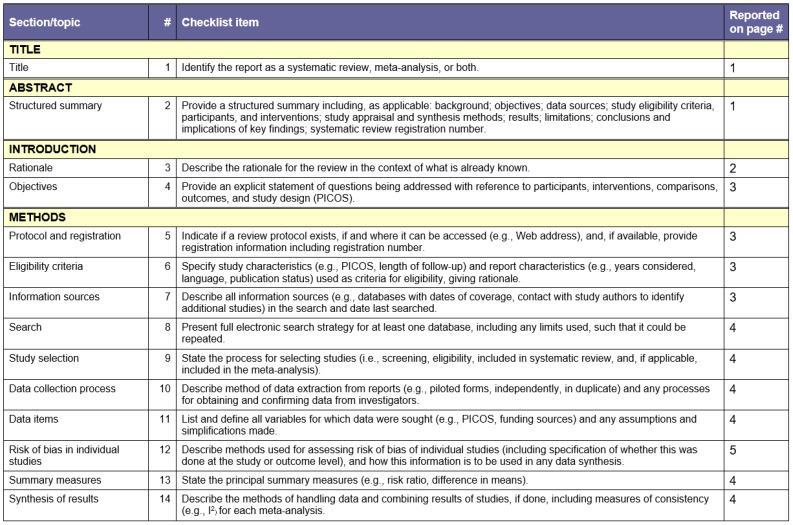

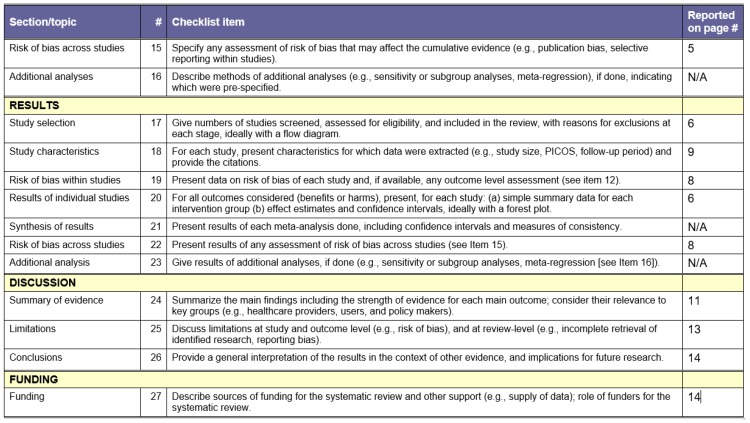
Preferred Reporting Items for Systematic Reviews and Meta-Analyses (PRISMA) checklist.

**Figure A2:**
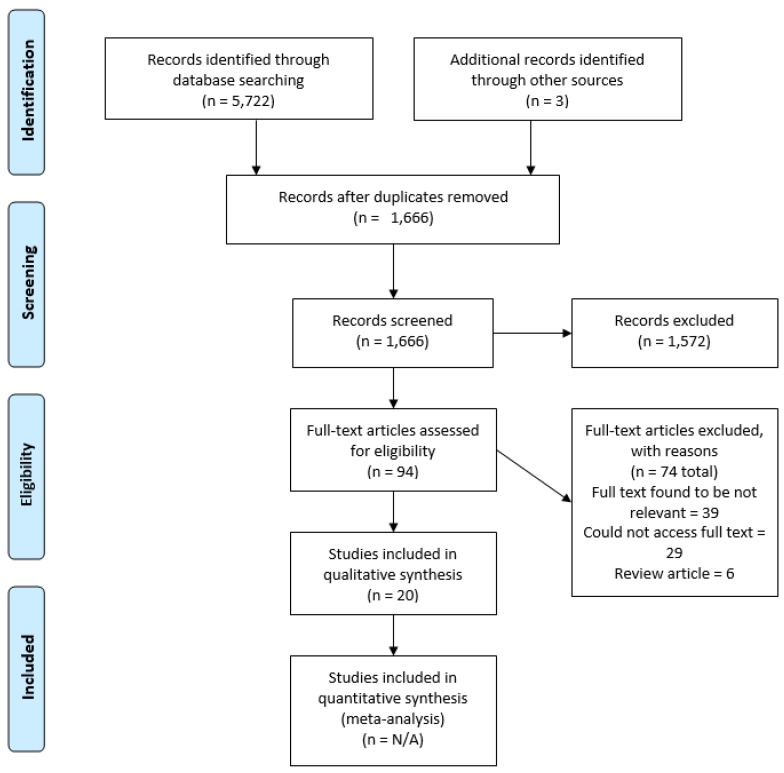
PRISMA flow diagram.
